# Beclin‐1 improves mitochondria‐associated membranes in the heart during endotoxemia

**DOI:** 10.1096/fba.2020-00039

**Published:** 2021-01-25

**Authors:** Yuxiao Sun, Ying Cai, Suhong Qian, Hellen Chiou, Qun S. Zang

**Affiliations:** ^1^ Department of Surgery University of Texas Southwestern Medical Center Dallas TX USA; ^2^ Department of Developmental Cell Biology Key Laboratory of Cell Biology China Medical University Shenyang Liaoning Province P. R. China; ^3^ Department of Surgery, Burn and Shock Trauma Research Institute Stritch School of Medicine Loyola University Chicago Health Sciences Division Maywood IL USA

**Keywords:** beclin‐1, cardiac dysfunction, LPS, MAMs, sepsis

## Abstract

Mitochondria‐associated membranes (MAMs) are essential to mitochondria. This study was to determine whether endotoxemia rearranges MAMs in the heart, and whether Beclin‐1 regulates this process. Wild‐type mice and mice with a cardiac‐specific overexpression of Beclin‐1 (*Becn1*‐*Tg*), or a heterozygous knockout of Beclin‐1 (*Becn1* ^+/−^) were given lipopolysaccharide (LPS) challenge. In the heart, the ultrastructure of MAMs was examined by electron microscopy and the histology evaluated by immunostaining. Additionally, MAMs were isolated by ultracentrifugation, and their content and function were quantified. The effects of Beclin‐1‐activating peptide (TB‐peptide) on MAMs were also examined. Data showed that endotoxemia decreased both the total mass and the function of MAMs, and these deficiencies became worse in *Becn1* ^+/−^ mice but were alleviated in *Becn1*‐*Tg* and TB‐peptide‐treated mice. Responses of myocardial MAMs to LPS and to TB‐peptide were additionally examined in AC16 human cardiomyocytes. *In vitro* findings recaptured the effects of LPS and TB‐peptide in cardiomyocytes; the challenge of LPS reduced the level and activity of MAMs, and TB‐peptide attenuated this defect. Together, the results suggest a new function of Beclin‐1 in improving cardiac MAMs during endotoxemia, providing a mechanism for the previously identified role of Beclin‐1 in protection of mitochondria and cardiac function.

**Abbreviations**
DAMPs: danger‐associated molecular patterns
ER: endoplasmic reticulum
GRP75: glucose‐regulated protein 75
LPS: lipopolysaccharide
MAMs: mitochondria‐associated membranes
mtDNA: mitochondrial DNA
mtROS: mitochondrial reactive oxygen species
PEN2: presenilin enhancer 2
TB‐peptide: Tat‐Beclin‐1 peptide
VDAC1: voltage‐dependent anion‐selective channel 1
WT: wild‐type

## 1 INTRODUCTION

Sepsis is a life‐threatening condition of organ dysfunction caused by a deregulated host response to infection.^1^ Despite improvements in antibiotic therapies and critical care techniques,^2^ sepsis remains a leading cause of death in critical care units,^1^ and its reported incidence is still increasing.^3^ In‐depth understanding of the pathological mechanisms and exploration of new therapeutic interventions for sepsis are in urgent need.

Cardiac dysfunction is a main predictor of poor outcomes in sepsis.^4^, ^5^, ^6^ In the heart, mitochondria comprise about 30% of myocardial volume.^7^ Research in the recent years have demonstrated that mitochondria are more than the powerhouses that generate ATP to meet energy demands. They are also involved in a plethora of important cellular processes such as inflammation, autophagy, cell death, apoptosis, and metabolism. Previously, our studies and others showed that sepsis triggers damage in mitochondria, resulting in a deficiency in ATP supply and an overproduction of mitochondria‐derived danger‐associated molecular patterns (DAMPs), such as mtROS and fragmented mtDNA.^8^, ^9^, ^10^ These harmful molecules released from damaged mitochondria become a driving force to exacerbate myocardial inflammation and cardiac dysfunction during sepsis.^9^, ^11^

Under normal physiology condition, the quality and quantity of mitochondria are tightly regulated by multiple aspects of cellular process, including mitochondrial biogenesis, the dynamic switch between fusion and fission, and recycling through mitophagy. Additional control over the mitochondrial population, distribution, and function is directly achieved by a subcellular domain termed mitochondria‐associated membranes (MAMs). MAMs are the regions of close physical connection between mitochondrial outer membrane and other intracellular membranes that are mainly from the endoplasmic reticulum (ER).^12^, ^13^, ^14^, ^15^, ^16^, ^17^ Mitochondrial physiology is regulated by MAMs particularly through mitochondria–ER communication in the transport of Ca^2+^ ^15^ and lipids.^18^ MAMs also function as a signaling hub harboring key molecules during protein sorting, ER stress, apoptosis, inflammation, and autophagy.^19^, ^20^ Aberrations in MAMs are linked to health problems with mitochondrial dysfunction as a major pathological component; the deregulation of MAMs was identified in preclinical and clinical samples of neurodegenerative diseases, diabetes, obesity, and infectious diseases.^21^, ^22^, ^23^, ^24^, ^25^, ^26^ In sepsis, a devastating deterioration in cardiac mitochondria, shown by a damaged integrity in structure, deficiency in function, and an overproduction of DAMPs, incites downstream inflammation.^8^, ^9^, ^10^, ^11^, ^27^ However, whether sepsis causes any defects in MAMs and whether MAMs play any pathological role in triggering sepsis‐induced mitochondria damage remain unknown.

Our laboratory recently investigated the role of Beclin‐1 in the heart during endotoxemia induced by lipopolysaccharide (LPS), a major molecule of pathogen‐associated molecular patterns released from gram‐negative bacteria.^27^ Beclin‐1 is a key autophagy initiation factor that is universally expressed.^28^, ^29^ We detected that the specific activation of Beclin‐1 improves cardiac function and limits myocardial inflammation during endotoxemia. This Beclin‐1‐dependent protection was associated with an improved quality control of mitochondria and a reduction in mitochondria‐derived DAMPs. Results also suggest that, mechanistically, Beclin‐1 removes damaged mitochondria by a selective activation of adaptive mitophagy in the heart under septic insults. As expected, the specific activation of Beclin‐1, either genetically or pharmacologically, significantly improved cardiac performance under the challenge by LPS,^27^ leading us to postulate that the targeted activation of autophagy factors is an effective approach to boost adaptive autophagic responses, and thus, improves outcomes in sepsis.

In the study summarized in this report, we extended our investigation to examine the potential role of MAMs in the mitochondrial pathology in sepsis and to determine whether Beclin‐1 possesses a regulatory power over this process. In a mouse model of endotoxemia, we obtained new findings showing that septic challenge by LPS incites losses in myocardial MAMs, and this damage was alleviated by the targeted activation of Beclin‐1 either genetically or pharmacologically.

## 2 MATERIALS AND METHODS

### 2.1 Experimental animals

Wild‐type (WT) C57BL/6 mice were obtained from Charles River laboratories and an in‐campus mouse breeding core facility at the University of Texas Southwestern Medical Center (UTSW). All animals were conditioned in‐house for 5‐6 days after arrival with commercial diet and tap water available at will. Mouse strains with a cardiac‐specific overexpression of Beclin‐1 under the α‐myosin heavy chain promoter (*Becn1*‐Tg)^30^ or haploinsufficient for *beclin*‐*1* (*Becn1* ^+/−^)^31^ were previously developed. Animal work descripted in this study was reviewed by and conducted under the oversight of UTSW institutional animal care and use committee and conformed to the National Research Council's “Guide for the Care and Use of Laboratory Animals” when establishing animal research standards.

LPS‐induced endotoxemia model: Male mice, 8‐12 weeks old, were weighed to determine the amount of LPS (#L3012; MilliporeSigma) required to achieve indicated doses and administered intraperitoneally (i.p.) in a volume of 100 μL per mouse. Sterile endotoxin‐free PBS was used as a vehicle control in sham groups. In some experiments, Beclin‐1‐activating peptide (TB‐peptide), synthesized according to a published sequence^32^ by NonoPep, was administered i.p. at a dose of 16 mg/kg in 100 μL of PBS 30 minutes post LPS challenge.

### 2.2 Culture of AC16 human cardiomyocyte cell line

AC 16 cells (#SCC109; MilliporeSigma) were incubated in medium Dulbecco's modified Eagle's medium/Nutrient Mixture F‐12 Ham (DMEM/F‐12) (#D6434; MilliporeSigma) containing 2 mmol/L L‐glutamine, 12.5% fetal bovine serum (FBS), and 1X penicillin‐streptomycin (#TMS‐002‐C, ES‐009‐B, and TMS‐AB2‐C; MilliporeSigma) at 37°C in a humidified incubator with 5% CO_2_. The medium was exchanged for fresh medium every 2‐3 days. After reaching to 90% confluency, cells were dissociated with trypsin‐EDTA (#SM‐2003‐C; MilliporeSigma) for further passage or experiments. In some experiments, cells were exposed to LPS and/or TB‐peptide in the conditions described in figures.

### 2.3 Preparation of cellular fractions

Heart tissues were harvested, washed in PBS, snap‐clamp frozen, and kept at −80°C until used. Procedures for the isolation of MAMs and mitochondria were performed at 4°C according to established protocols^33^, ^34^ with minor modifications. Briefly, tissue pieces of one mouse heart were homogenized in 1 mL IB_heart_ buffer (220 mmol/L mannitol, 70 mmol/L sucrose, 10 mmol/L HEPES, and 1 mmol/L EGTA, pH7.4) using a Potter‐Elvehjem PTFE pestle and glass homogenizer (#P7734; MilliporeSigma), which was driven by a stirrer motor with electronic speed controller (#EW‐04369‐10; Cole‐Palmer), by 40 strokes at a speed of 1500 rpm followed by another 40 strokes at 800 rpm. Crude mitochondrial fractions were then obtained by differential centrifugation in the following two steps. First, the homogenized heart lysates were subjected to twice‐repeated centrifugation at 740 *g* for 5 minutes to remove unbroken cells and nuclei. Second, the supernatant mixtures were centrifuged at 9000 *g* for 10 minutes to collect pellets. These pellets were then resuspended in freshly prepared mitochondria‐resuspension buffer (MRB; 250 mmol/L mannitol, 5 mmol/L HEPES, and 0.5 mmol/L EGTA, pH7.4) and subjected to twice‐repeated centrifugation at 10 000 *g* for 10 minutes to collect crude mitochondria. The crude mitochondria pellets were then resuspended in MRB at the ratio of 0.5 mL MRB per heart and subjected to ultracentrifugation (Sorvall MX 120 Plus Micro‐Ultracentrifuge with rotor S50‐ST, #50135645; Thermo Scientific) to isolate MAMs and pure mitochondria (PM) by the following three steps. First, in each 7 mL ultracentrifuge tube, 6 mL of freshly made percoll medium (225 mmol/L mannitol, 25 mmol/L HEPES (pH7.4), 1 mmol/L EGTA, and 30% percoll (v/v)) was layered with 0.5 mL of crude mitochondria resuspension and 0.5 mL of MRB, from the bottom to the top, and centrifuged at 95 000 *g* for 30 minutes. Fractions of mitochondria, dense bands located approximately at the bottom, and MAMs, diffused white bands located above the mitochondria, were collected. Second, the collected bands of mitochondria and MAMs were diluted 10 times with MRB and further centrifuged at 6300 *g* for 10 minutes. Third, mixtures of MAMs bands and mitochondria bands were centrifuged at 100 000 *g* for 1 hour. For fractions of MAMs, the pellets were collected and stored at −80°C until used. For fractions of mitochondria, the pellets were collected and resuspended with MRB again, followed by another two washes by centrifugation at 6300 *g* for 10 minutes, and the PM pellets were then collected and stored at −80°C until used. All chemicals were purchased from MilliporeSigma.

### 2.4 Electron microscopy

Mid‐sections of the left ventricular wall tissue of harvested hearts were cut into small pieces, about 13 mm in size, and fixed in buffer of 2.5% glutaraldehyde/0.1 mol/L Na cacodylate, pH7.4. Sections (75‐80 nm) were cut using a Leica Ultramicrotome and examined under electron microscopy.

### 2.5 Immunostaining

Fresh heart tissues were perfused in PBS, followed by fixation in 4% paraformaldehyde, and then left in fixation buffer for 24 hours at 4°C. For dehydration, fixed tissues were first transferred to 10% sucrose/PBS for 24 hours, then to 18% sucrose/PBS for another 24 hours, and both steps were performed at 4°C. Tissue samples were embedded in OCT, sectioned at 8 μm, air‐dried and stored at −80°C until used. For staining, frozen slides were then thawed, rehydrated, and subjected to immunochemistry. Slides were blocked with 3% donkey serum/PBS, stained with a goat polyclonal calreticulin antibody (1:50) and a rabbit polyclonal monoclonal VDAC1 antibody (1:50; #ab4109 and ab15895; Abcam, Cambridge, MA) for 1 hour at room temperature, followed by another 1 hour incubation with Alexa Fluor 88‐labeled donkey anti‐rabbit IgG (1:500) and Cy3‐labeled donkey anti‐goat IgG (1:500; #711‐546‐152 and 705‐166‐147; Jackson ImmunoResearch). In some experiments, tissue slides were co‐stained with VDAC1 and Nile red to visualize mitochondria and lipid contents. Slides were incubated with anti‐VDAC1 as above, and followed by another 1 hour incubation with biotinlyated goat anti‐rabbit IgG (1:400; ab64256; Abcam). The antibody signals were visualized by adding horseradish peroxidase (HRP) substrate (ImmPACT DAB substrate; SK4105; Vectror Lab), and the reaction was stopped using water after a 5‐minute incubation. Each slide was then added a drop of Nile red (1:200 in 75% glycerol, diluted from stock solution of 500ug/ml in acetone; #109123; MilliporeSigma), incubated for 5 minutes, and sealed with coverslip. All slides were examined under a Nikon Eclipse Ti‐E inverted microscope at 40× magnification.

When immunostaining was performed in AC16 cells, cells were seeded on the gelatin‐coated coverslips till 70%‐80% confluency. After being washed with PBS, fixed with 4% paraformaldehyde, and permeabilized with 0.2% triton X‐100/PBS, coverslips were blocked with 3% donkey serum/2% BSA/PBS, stained with a rabbit polyclonal Tom 20 antibody (1:50) and a mouse monoclonal antibody against calnexin (1:20; #SC11415 and SC46669; Santa Cruz Biotechnology) for 1 hour at room temperature, followed by another 1 hour incubation with Cy3‐labeled donkey anti‐rabbit‐IgG (1:500) and Alexa Fluor 88‐labeled donkey anti‐mouse IgG (1:500; #711‐166‐152 and 715‐546‐150; Jackson ImmunoResearch). The coverslips were then examined under a Nikon Eclipse Ti‐E inverted microscope at 40x magnification.

### 2.6 Western blots

Prepared SDS‐PAGE protein samples were loaded to and run on 15% SDS‐PAGE gels, and transferred to PVDF membranes. Membranes were blocked with 5% nonfat milk‐PBS at room temperature for 1 hour and subsequently probed with one of the following antibodies according to experiments: GAPDH (#MAB374; Millipore), LC3 and p62 antibodies (#4108 and 5114; Cell Signaling), VDAC1, cytochrome C, FALC4, and PEN2 (#ab14734, ab110325, ab155282, and ab18189; Abcam), and Grp75 (#sc‐133173; Santa Cruz Biotechnology). The membranes were then rinsed and incubated with corresponding horseradish peroxidase‐conjugated secondary antibodies (#170‐6515 and 170‐6516; Bio‐Rad). Antibody dilutions and incubation time were according to manufacturer's instructions. At the end, membranes were rinsed and bound antibodies were detected by using SuperSignal West Pico Chemiluminescent Substrate (#34077; ThermoFisher Scientific).

### 2.7 Quantification of phospholipids

Levels of phospholipids in mitochondria were measured with a phospholipid assay kit (#MAK122; MilliporeSigma). Most phospholipids contain one diglyceride, a phosphate group, and one choline. This assay was designed to quantify choline‐containing phospholipids in samples. According to the manufacturer's protocol, fractions of mitochondria were diluted to 1‐2.5 μg protein per assay using the assay buffer provided. Each reaction mix was set up by adding a prepared sample or standard to phospholipid D that degrades phospholipids to release choline. The amount of choline was determined with choline oxidase and an H_2_O_2_ specific dye. A colorimetric reading at wavelength 570 nm was proportional to the phospholipid concentration in the sample. Final results were calculated according to the standard curve and normalized by protein amount per sample, and measurements were performed in triplicates.

### 2.8 Cytotoxicity assay

Cytotoxicity of AC16 cells in response to a treatment was estimated by a lactate dehydrogenase (LDH) assay kit (#ab65393; Abcam). Cells were washed and collected with fresh culture medium, and seeded at density 1.75 × 10^4^/100 ul/well on 96‐well plates. After settled on plates for overnight in a tissue culture incubator, cells were treated at conditions according to the experimental design, followed by the measurement of LDH, an enzyme marker of plasma membrane damage, according to the manufacturer's protocol. Briefly, cells were precipitated by centrifugation at 600 g for 10 minutes, cell medium was transferred to an optically clear 96‐well plate and mixed with reaction buffer for 10 minutes, and the absorbance at wavelength 450 nm was measured. All measurements were performed in triplicates.

### 2.9 Statistical analysis

All data were expressed as mean ±SEM of at least three independent experiments using 4‐6 animals/group. The two tailed unpaired *t* tests were used for comparisons between groups. Differences were considered statistically significant as *p* ≤ .05.

## 3 RESULTS

### 3.1 LPS‐dependent reduction in cardiac MAMs and the role of Beclin‐1

Recently, we discovered that enhancing Beclin‐1‐dependent autophagy attenuates mitochondrial damage in the heart during endotoxemia.^27^ Since MAMs play essential roles to keep the fitness of mitochondrial health, we went on to determine whether LPS challenge affects the physiology of cardiac MAMs and whether Beclin‐1 regulates this process.

**FIGURE 1:**
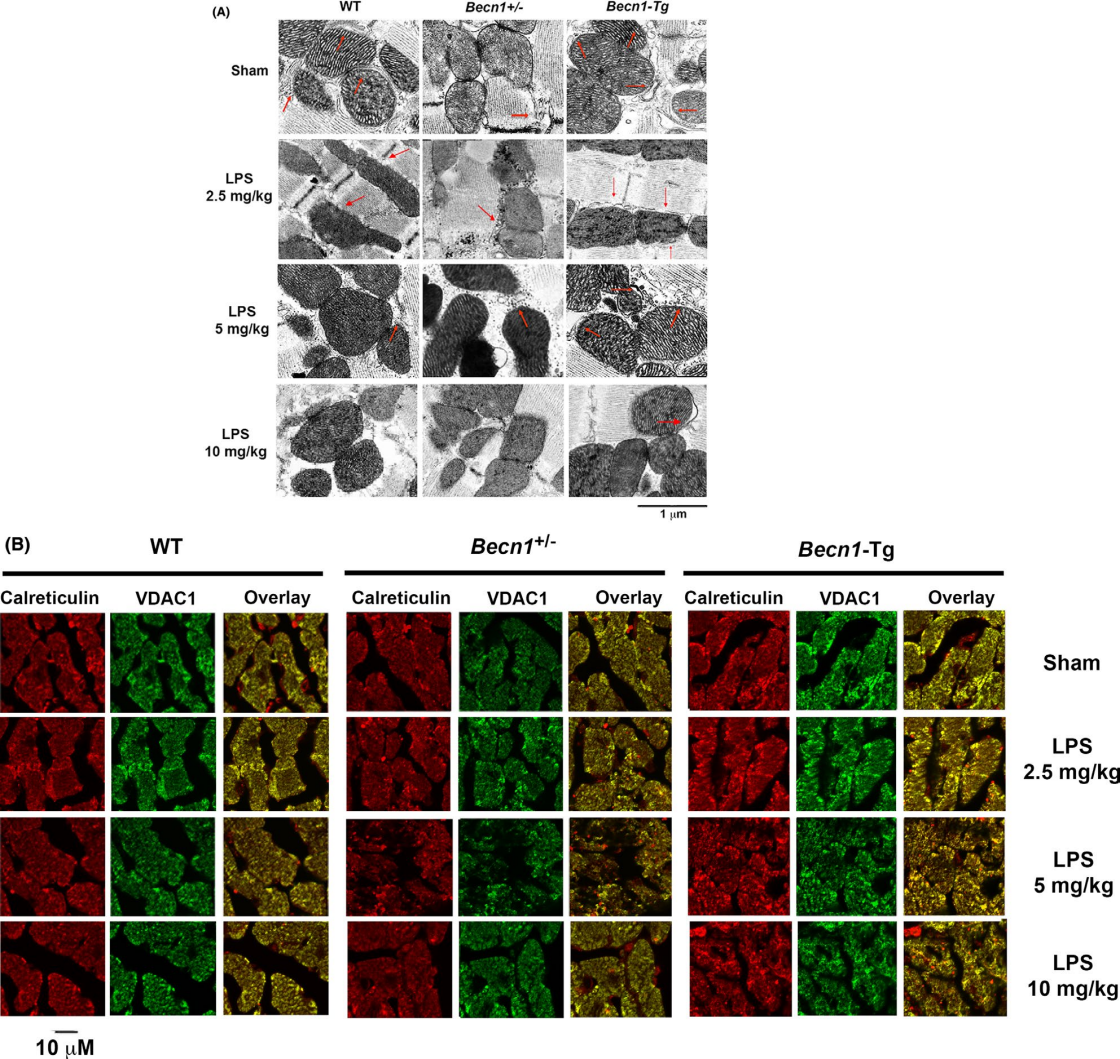
LPS‐induced changes in the structure of cardiac MAMs in WT, *Becn1*‐Tg, and *Becn1* ^+/−^ mice. Mice were given indicated doses of LPS via i.p., and heart tissues were harvested 18 hours later. A, Ultrastructure of mitochondria and MAMs was observed by transmission electron microscope. Red arrows indicate the structure of MAMs. B, Heart tissue sections were co‐immune‐stained with the ER marker calreticulin (red) and mitochondria marker VDAC1 (green). Overlay color in yellow indicates the levels of MAMs. All images are representative of n ≥ 3 animals per group

We compared the WT mice with mouse lines carrying either a cardiac‐specific overexpression of Beclin‐1 (*Becn1*‐Tg) or haploinsufficiency for *beclin‐1* (*Becn1* ^+/−^). Mice were given challenge of LPS at indicated doses, and the heart tissue was collected 18 hours post LPS challenge for the following examinations and. Electron microscopy was used to compare the morphology of mitochondria and their surrounding MAMs in the left ventricular wall of hearts from WT, *Becn1*‐Tg and *Becn1* ^+/−^ mice at baseline and following the challenge by LPS at 2.5, 5, and 10 mg/kg. As shown in Figure 1A, we observed LPS dose‐dependent decrease in the degree of MAM formation, indicated by red arrows, in WT mice. This decrease was associated with a loss of clearly defined cristae structures of the inner mitochondrial membrane, which showed a damage in mitochondria. These disruptions were more severe in the *Becn1* ^+/−^ mice; the reduction in MAMs was evident even in the sham group compared with its WT counterpart. On the contrary, *Becn1*‐Tg mice given LPS showed near‐to‐normal structures of MAMs and mitochondria in the heart.

The heart tissue was also examined by immunostaining using VDAC1, a marker of mitochondria, and calreticulin, a maker of ER, for the purpose of detecting the degree of mitochondria–ER tethering that indicates MAMs. As presented in Figure 1B, comparisons between sham and LPS‐treated mice revealed that LPS caused a gradual reduction in the formation of MAMs, shown by the decreased amount of overlay between VDAC1 and calreticulin the in color yellow, in all three strains. Further, an increase in the levels of MAMs in *Becn1*‐Tg mice but a decrease in *Becn1* ^+/−^ mice were also evident. Taken together, these results suggest: (1) endotoxemia shock causes a dose‐dependent decrease in MAM formation, and (2) Beclin‐1 has a function in the regulation of MAMs in the heart during endotoxemia.

### 3.2 LPS‐dependent decrease in cardiac lipid accumulation and the role of beclin‐1

**FIGURE 2:**
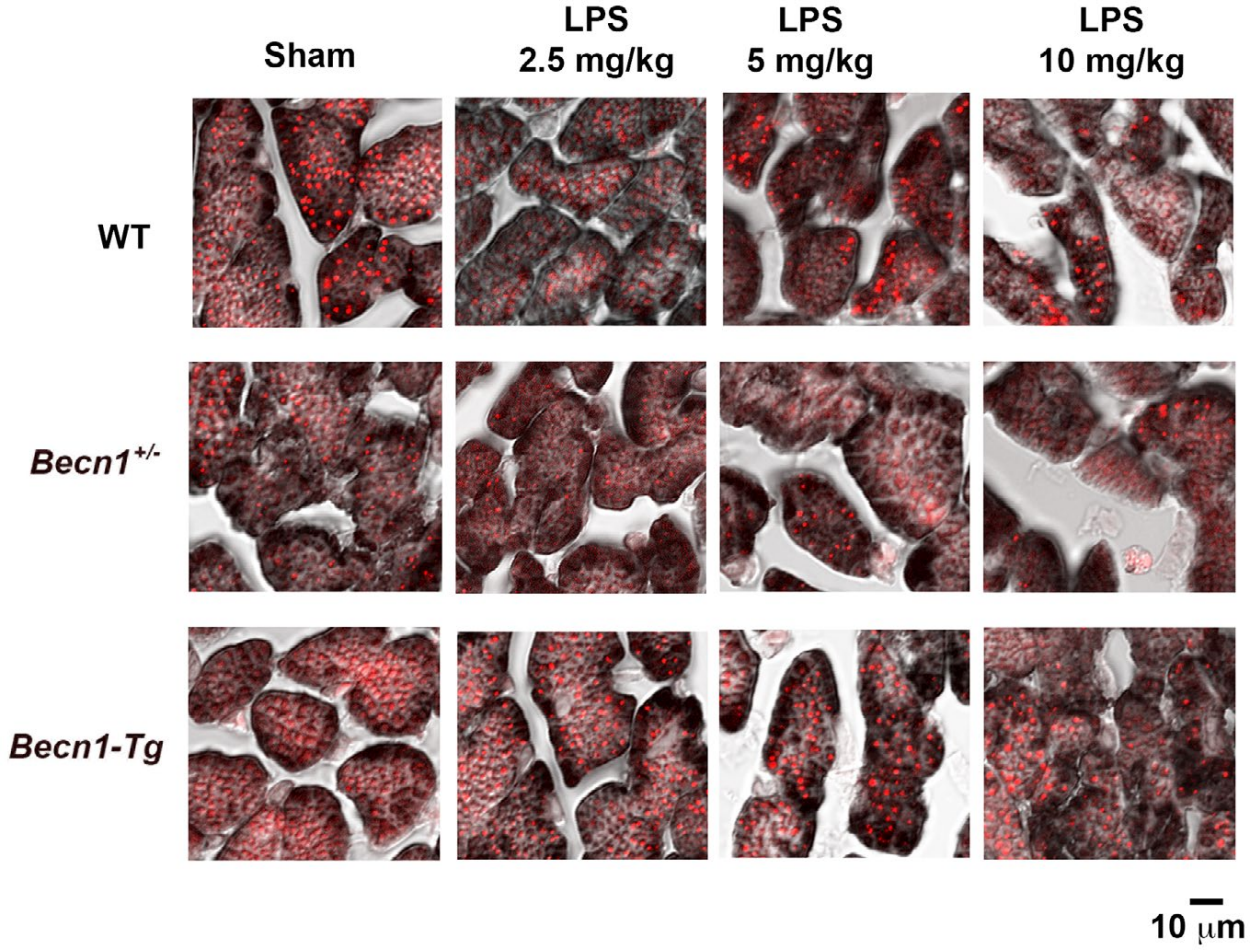
LPS‐induced changes in mitochondria and lipid contents in WT, *Becn1*‐Tg, and *Becn1* ^+/−^ mice. Mice were given indicated doses of LPS via i.p., and heart tissues were harvested 18 hours later. Heart tissue sections were co‐stained with mitochondria marker VDAC1 (brown) and lipid marker Nile red (red). All images are representative of n ≥ 3 animals per group

While in charge of the transport of calcium and lipids between mitochondria and ER, MAMs serve as a special hub for enzymes of lipid trafficking and synthesis, such as acyl‐CoA: cholesterol acyltransferase/sterol O‐acyltransferase 1 (ACAT1/SOAT1), diacylglycerol O‐acyltransferase 2 (DGAT2), PS synthases 1 and 2 (PSS1 and PSS2), phosphatidylethanolamine N‐methyltransferase 2 (PEMT2), and fatty acid CoA ligase 4 (FACL4/ACS4).^35^, ^36^, ^37^, ^38^ In addition, the unique heterogeneity of the MAM structure requires lipid constituents such as cholesterol and sphingolipids to support the formation of MAMs and to fortify the membrane thickness of MAMs. As such, MAMs were reported to be fortified with cholesterol and sphingolipids, which increase their thickness.^39^, ^40^ Therefore, the level of cardiac lipid accumulation is closely related to the proper formation and physiology of MAMs in the heart tissue. Our previous research showed that LPS decreases mitochondrial mass and function in the heart.^27^ In this report, we compared lipid levels in relative to mitochondrial mass in the heart tissue of WT, *Becn1*‐Tg, and *Becn1* ^+/−^ mice. Tissue slides were co‐stained with lipid‐specific florescent dye Nile red (color red) and mitochondrial marker VDAC1 (brown). As shown in Figure 2, LPS challenge induced a dose‐dependent decrease in the ratio of lipid vs mitochondria, and this decrease was evidently attenuated in the strain of *Becn1*‐Tg mice. Significantly, downregulation of Beclin‐1 in *Becn1* ^+/−^ mice severely suppressed lipid accumulation in the heart, even in the group of sham controls. This observation is consistent with a previously published study showing LPS‐stimulated reduction in cardiac lipid accumulation in mice.^41^ It further suggests that, under both physiological condition and physiological condition of endotoxemia, Beclin‐1 signaling is required to retain lipid levels in the heart, which is likely at least one of the mechanisms underlying Beclin‐1‐dependent support of MAMs.

Our previous investigation demonstrated that LPS challenge at 5 mg/kg caused disrupted cardiac contractility in WT mice but not in *Becn1*‐Tg mice. However, in *Becn1* ^+/−^ mice, cardiac dysfunction started showing when given LPS at 2.5 mg/kg and progressed worsen at 5 mg/kg.^27^ For the rest of the report, we chose to examine the features of cardiac MAMs when mice were challenged by LPS at 5 mg/kg, at which dose the most significant difference in cardiac performance was shown by the three strains of mice.^27^

### 3.3 BECLIN‐1 preserves the mass and function of cardiac MAMs during endotoxemia

**FIGURE 3:**
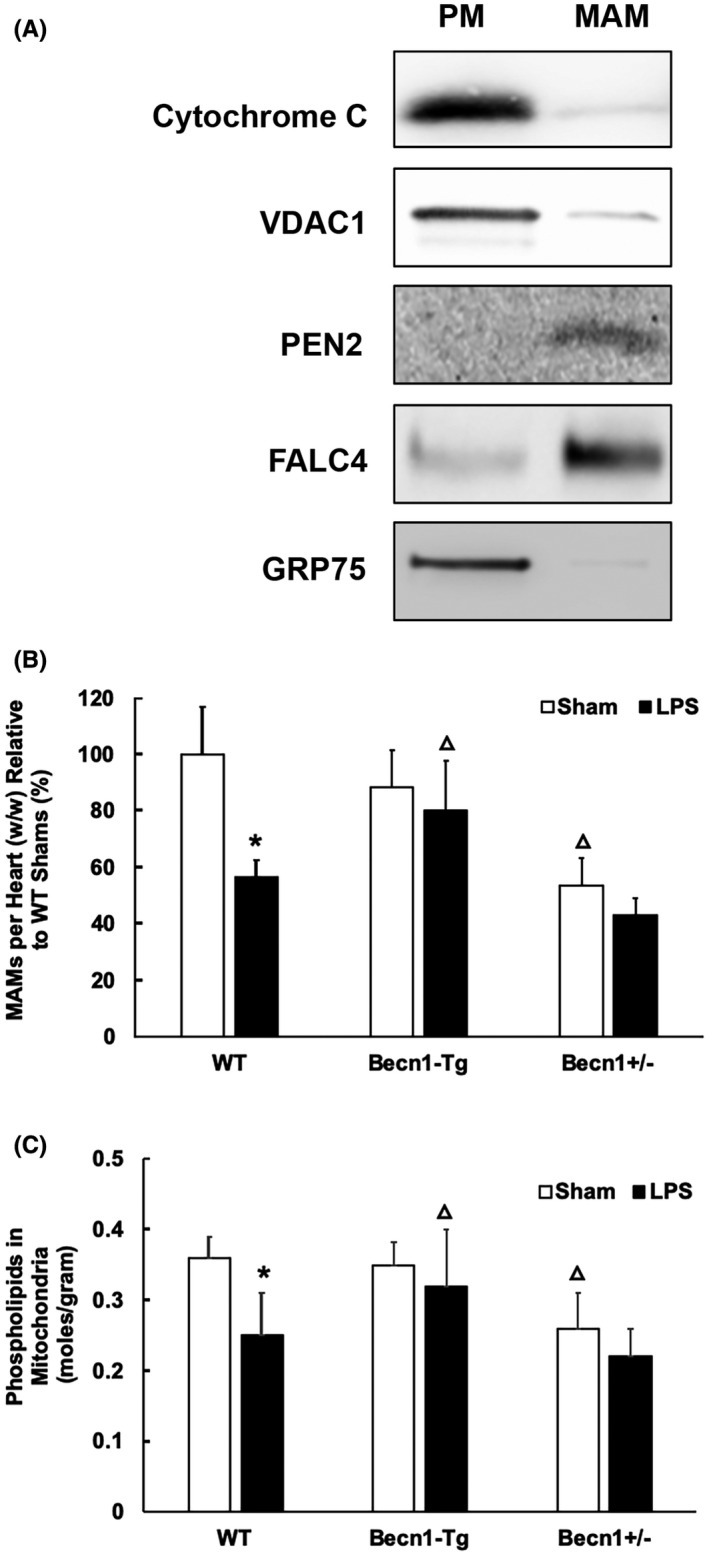
LPS‐induced changes in the mass and function of cardiac MAMs in WT, *Becn1*‐Tg, and *Becn1* ^+/−^ mice. Mice were given 5 mg/kg LPS via i.p., and heart tissues were harvested 18 hours later. Fractions of pure mitochondria (PM) and MAMs were prepared by ultracentrifugation from the heart tissue. A, Successful isolation of MAMs and PM from the hearts of WT mice was demonstrated by Western blots detecting marker proteins of mitochondria and ER. B, The amount of each fraction of MAMs were measured and results were normalized by tissue weight. C, Levels of phospholipids in mitochondrial fractions were quantified and results were normalized by the amount of protein. In B and C, values are means ±SEM. Significant differences are shown as * for sham vs LPS‐treated and Δ for WT vs *Becn1*‐Tg or *Becn1* ^+/−^ groups (*P* < .05, n = 4‐6, unpaired *t* test)

To directly examine whether the insult of LPS challenge alters MAMs in the heart, and whether Beclin‐1 has any regulation on this response, we quantified and compared the amount and function of MAMs in the heart tissue of WT, *Becn1*‐Tg, and *Becn1* ^+/−^ mice at baseline and following challenge by LPS. First, we validated the procedure of isolating MAMs and PM fractions by ultracentrifugation based on published protocols,^32^, ^33^ as described in the section of Methods and Materials section. The successful separation of MAMs from mitochondria was demonstrated by the detection of specific markers in each isolation (Figure 3A). Cytochrome C, an enzyme located in the mitochondrial intermembrane space, was exclusively located in PM but not in MAMs. Mitochondrial outer membrane protein VADC1 and mitochondrial chaperone GRP75, which both form complexes with partners proteins on the ER for mitochondria–ER tethering, were detected primarily in PM. PEN2, a subunit of gamma secretase complex located on the ER membranes, was found in MAMs but not in PM. Fatty acid CoA ligase 4 (FACL4), an enzyme enriched in MAMs to facilitate lipid metabolism, was detected mainly in MAMs and not in PM. As summarized in Figure 3B, the amount of MAM isolation in the heart was quantified based on tissue weight. A toxic dose of LPS, 5 mg/kg, triggered a significant, over 40% drop in the quantity of MAMs in WT mice, but this response was sufficiently alleviated by the overexpression of Beclin‐1 in *Becn1*‐*Tg* mice. On the contrary, the downregulation of Beclin‐1 in *Becn1* ^+/−^ mice resulted in a 40% decrease in MAMs at the baseline and an additional 10% in response to LPS.

A fundamental function of MAMs is to coordinate the synthesis and transport of phospholipids to other organelles such as mitochondria.^42^ Because mitochondria are unable to synthesize phospholipids de novo and rely on the ER as the sole supplier,^43^, ^44^ mitochondrial phospholipid levels indicate the function of MAMs.^45^ By the quantification of total phospholipids in mitochondrial fractions, we found that LPS caused a ~30% decrease in MAM function in the heart; however, this deficiency was not detected in *Becn1*‐*Tg* mice (Figure 3C). In *Becn1* ^+/−^ mice, the baseline level of cardiac MAMs was about 25% lower than that of the WT mice, and LPS challenge induced a further slight reduction but not one that was statistically significant. Overall, these observations suggest that LPS induces deficiencies in cardiac MAMs, and Beclin‐1 is able to control this deterioration process during endotoxemia.

### 3.4 BECLIN‐1‐activating peptide has an effect to protect cardiac MAMs from endotoxemia

**FIGURE 4:**
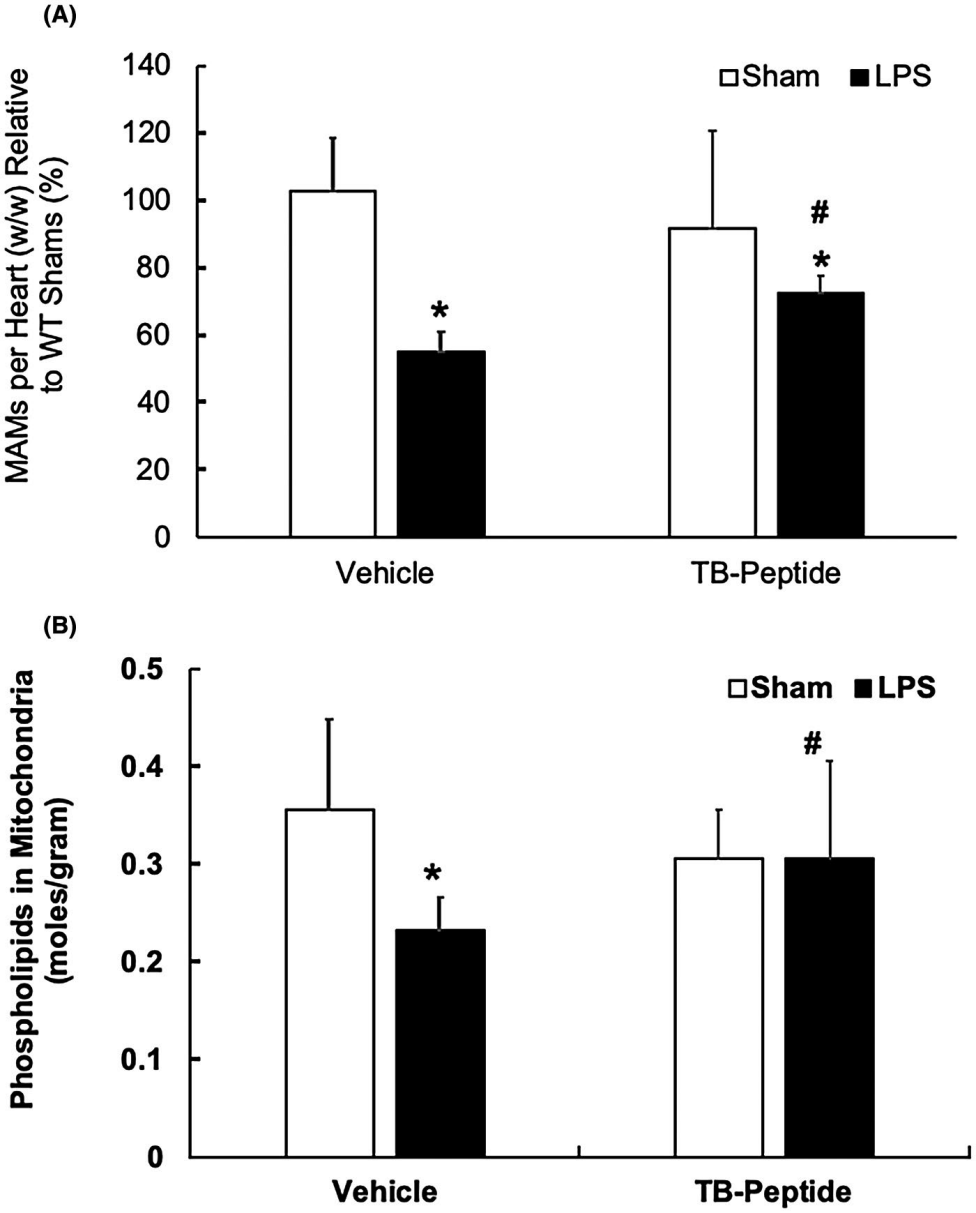
Effects of TB‐peptide on cardiac MAMs in LPS‐challenged mice. WT mice were given 5 mg/kg LPS i.p., then TB‐peptide, 16 mg/kg, was administered i.p. 30 minutes post LPS challenge. Heart tissues were collected 18 h post LPS challenge, and fractions of mitochondria and MAMs were prepared by ultracentrifugation. A, The amount of each fraction of MAMs was measured and results were normalized by tissue weight. B, Levels of phospholipids in mitochondrial fractions were quantified and results were normalized by the amount of protein. All values are means ±SEM. Significant differences are shown as * for sham vs LPS‐treated and # for without vs with TB‐peptide (*P* < .05, n = 4‐6, unpaired *t* test)

A cell‐permeable, autophagy‐inducing Tat‐Beclin‐1 peptide, TB‐peptide, were previously demonstrated therapeutic benefits in disease models related to reducing viral infection,^46^ improving cardiac performance during pressure overload,^47^ and enhancing the effectiveness of chemotherapy.^32^ We have also shown that this peptide improves heart function during endotoxemia.^27^ To examine whether this pharmacological approach has a potential to alleviate the damage of MAMs in endotoxemia, we compared MAM mass and function in the heart tissue of LPS‐challenged mice with or without TB‐peptide. As shown in Figure 4A, the administration of TB‐peptide following LPS challenge attenuated an LPS‐induced decline in MAM mass; the treatment minimized the LPS‐induced reduction in MAM quantity from 50% to ~20% compared to the sham group. Measurement of phospholipids showed that TB‐peptide significantly improved the function of MAMs in the heart tissue in endotoxemia (Figure 4B).

### 3.5 TB‐peptide protects mams in human cardiomyocyte challenged by LPS

**FIGURE 5:**
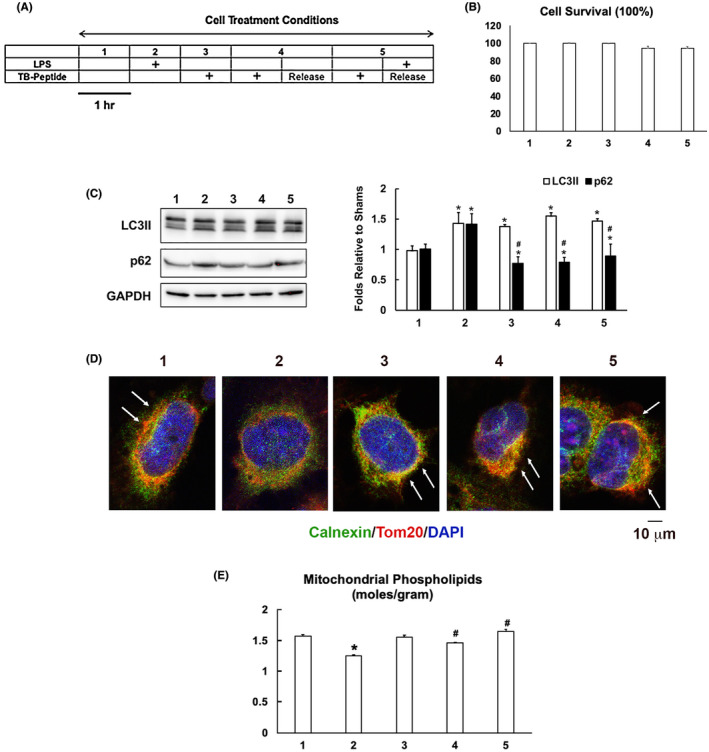
TB‐peptide promotes autophagy and protects MAMs in human cardiomyocyte challenged by LPS. A, AC16 cells were cultured until 80%‐90% confluency and treated with the conditions as illustrated. B, The percentage of cell survival was calculated based on the analysis of cytotoxicity. C, Levels of LC3II and p62 in cell lysates were examined by western blot and quantified by densitometry. GAPDH was used as a loading control. D, Cells were co‐immune‐stained with the ER marker calnexin (green) and mitochondria marker Tom 20 (red). Staining of nucleus by DAPI is shown in blue. Overlay areas of mitochondria–ER contact shown in the color yellow indicates the levels of MAMs and are labeled with white arrows. All images are representative of ≥3 independent experiments. E, Levels of phospholipids in mitochondrial fractions were quantified and results were normalized by the amount of protein. All values are means ±SEM. Significant differences are shown as * for sham vs LPS‐treated and # for without vs with TB‐peptide (*p* < .05, n = 3‐5, unpaired *t* test)

To further elucidate whether Beclin‐1 regulates MAMs in myocardium, we examined the effects of TB‐peptide on MAMs in human cardiomyocyte AC16 cells under challenge by LPS. We titrated various treatment conditions of LPS and TB‐peptide and chose those under which cell toxicity was undetectable. As illustrated in Figure 4A, cells were treated under the following five conditions: (a) control; (b) LPS 185 ng/mL for 1 hour; (c) TB‐peptide 5 μmol/L for 1 hour; (d) TB‐peptide 5 μmol/L for 1 hour, after which cells were washed and cultured in control medium for 1 hour (treatment released); (e) TB‐peptide 5 μmol/L for 1 hour after which treatment was replaced with an LPS 185 ng/mL challenge for 1 hour. In condition (f), cells were pretreated with TB‐peptide prior to LPS challenge, and the potential interference between the two reagents was avoided. These treatment conditions had no impact on cell survival (Figure 5B). To evaluate the peptide's effect on enhancing autophagy, cell lysates were subjected to the examination of LC3II, a marker of autophagosome formation during autophagy initiation, and p62/SQSTM1, a polyubiquitin‐binding autophagy adaptor protein which decrease marks lysosomal degradation at the later phase of autophagy flux, by western blot. As shown in Figure 5C, LPS challenge stimulated an accumulation of both LC3II and p62, suggesting that LPS at the indicated dosage causes a blockage in the flow of autophagy flux. However, the treatment with TB‐peptide promoted autophagic response, as shown by a significant increase in LC3II but decrease in p62. In the cells under the same conditions, double‐immunostaining using mitochondrial marker Tom 20 and ER maker calnexin visualized the areas of MAMs, shown by mitochondria–ER overlay in the color yellow and indicated by white arrows (Figure 5D). The result indicates that LPS challenge caused a reduction in MAMs, and TB‐peptide promoted the formation of MAMs under the normal condition and preserved the amount of MAMs in response to LPS. Moreover, TB‐peptide demonstrated a similar effect when the function of MAMs was measured by the levels of mitochondrial phospholipids (Figure 5E). These in vitro data confirmed what was observed in vivo, showing that the activation of the Beclin‐1 pathway preserved MAMs in myocardium in response to endotoxemia.

## 4 DISCUSSION

Studies of both clinical samples and animal models demonstrated that damage in mitochondria is one of the critical pathogenesis factors in inducing inflammation and multiorgan failure including cardiomyopathy in sepsis.^8^, ^9^, ^10^, ^11^ Recently, our investigation showed that the targeted activation of the autophagy initiation factor Beclin‐1 improves the quality control of mitochondria in the heart tissue and sequentially attenuates cardiac dysfunction in response to endotoxemia.^27^ Since mitochondrial physiology is tightly supported by the surrounding subcellular domain of MAMs, we therefore went on to examine whether LPS challenge causes any deficiencies in myocardial MAMs and whether Beclin‐1 is capable of controlling this process. Here we reported results obtained from a mouse model of endotoxemia showing that a toxic challenge of LPS produced a profound loss in both the mass and the function of MAMs in the heart (Figures 1, 2, 3). These defects were lessened in animals with a cardiac‐specific overexpression of Beclin‐1 but aggravated in those haploinsufficient for *beclin‐1*, strongly indicating a regulatory role for Beclin‐1 in maintaining proper MAMs in the cardiac tissue. Furthermore, the pharmacological activation of Beclin‐1 by TB‐peptide showed a beneficial effect by preserving MAM mass and function in those mice undergoing endotoxemia (Figure 4). In vitro studies using human cardiomyocyte AC16 cells further complemented the above *in vivo* observations; TB‐peptide was able to stimulate autophagy and to protect MAMs in response to challenge by LPS (Figure 5). Together, these data provide evidence indicating that endotoxemia during septic shock triggers a severe damage in cardiac MAMs, which is hypothesized as one of the causative mechanisms for inducing mitochondrial deficiencies, the accumulation of mitochondrial DAMPs, and sequential cardiomyopathy in sepsis. The data also suggest that pharmacological approaches that activate Beclin‐1 signaling may hold a therapeutic potential to preserve MAM properties during endotoxemia.

While our previous investigation suggests that Beclin‐1 protects cardiac mitochondria via the selection of a certain mitophagy pathway,^27^ data presented here support that the mechanism of this Beclin‐1‐dependent protection may go beyond mitophagy and involve MAMs. In this report, we found that LPS triggers a dose‐dependent reduction in the content of MAMs, starting from the lowest tested dosage of 2.5 mg/kg (Figure 1). Our previous evaluation of myocardial mitochondria status in the same model showed that LPS‐induced decrease in mitochondrial mass did not occur until the LPS challenge reached to a physiologically toxic level at 5 mg/kg.^27^ These data suggest that, compared with mitochondria, features of MAMs are more sensitive to the challenge of endotoxemia. Thus, it is hypothesized that an acute septic challenge, simulated by LPS, transduces a damage signal to the myocytes and causes a decrease in MAM content, which then results in a reduced mitochondrial mass. Our data further showed that enhancing Beclin‐1 signal provides a capability to mitigate LPS‐induced decrease in MAMs. Additionally, as previously shown, in the heart of LPS‐challenged mice, the upregulation of Beclin‐1 accumulates PTEN‐induced putative kinase 1 (PINK1) and the E3 ubiquitin ligase Parkin in mitochondria, while strongly suppressing other mitophagy mediators such as the receptor protein BNIP3L and BNIP3,^27^ suggesting that Beclin‐1 achieves the elimination of dysfunctional mitochondria by selecting PINK1‐Parkin mitophagy instead of a bulk induction of all types of mitophagy. Data presented here obtained both *in vitro* and *in vivo* suggest that Beclin‐1 has a new function to maintain proper MAMs in the heart, and a specific activation of Beclin‐1 signaling can attenuate an LPS‐induced decline in MAM quality and quantity during endotoxemia. Other published *in vitro* studies using cultured cell lines such as HEK293 and Hela showed that force‐expressed Beclin‐1 and PINK1 were accumulated in MAMs and both were required for the formation of mitochondria–ER tethering, suggesting a feedforward crosstalk between mitophagy and MAMs.^48^, ^49^, ^50^ Thus, based on our current studies, we speculate that Beclin‐1 attenuates LPS‐induced mitochondrial deficiencies in the heart through its actions both in preserving MAMs and in inducing mitophagy. Whether the induction of PINK1‐Parkin mitophagy is dependent on the integrity of MAMs in Beclin‐1 signaling, or vice versa, would be important questions to pursue in follow‐up studies.

It remains important to address whether Beclin‐1‐mediated regulation of MAMs is an autophagy‐dependent or ‐independent mechanism. Though the most recognized function of Beclin‐1 is to initiate autophagy, the function of Beclin‐1 in non‐autophagy pathways cannot be neglected. Beclin‐1 is a component of multiprotein complex of phosphatidylinositol‐3‐kinase (PI3K) class III, generating phosphatidylinositol‐3‐phosphate for membrane trafficking.^51^ When together with Atg14, Beclin‐1 is involved in inducing membrane elongation and maturation of both autophagosomes and phagosomes for autophagy. Alternatively, Beclin‐1 may also interact with UVRAG to stimulate vesicle formation that feeds endocytic trafficking.^52^ The autophagy‐independent function of Beclin‐1 in endosomal membrane dynamics was shown to be necessary in acquiring neuron viability and in facilitating skin development in animal models.^53^, ^54^ Since Beclin‐1 was detected in MAMs,^49^ it might directly support the formation of MAMs via interacting with other membrane trafficking factors such as UVRAG or the like. Further dissecting the autophagy‐dependent and ‐independent mechanisms of Beclin‐1 in MAMs and mitochondria can be accomplished by utilizing genetic models carrying a knockout and/or transgenic expression of specific downstream factors of Beclin‐1, such as Atg14 and/or UVRAG.

The regulation of mitochondria homeostasis by MAMs may also involve a dynamic collection of signaling molecules located at MAMs. Molecules recruited to MAMs or transported out of MAMs change constantly in response to physiological conditions or pathological challenges. A recently published proteomic study identified over 1200 proteins associated with MAMs in mouse tissue of brain and liver.^19^ Identified core components of MAMs include necessary factors necessary for sufficient mitochondria–ER communication in the transport of Ca^2+^ ^15^ and lipids,^18^ as well as those that support mitochondrial morphology. Many others core components belong to a variety of categories including factors in autophagy, inflammation, apoptosis, ER stress, and metabolism.^55^, ^56^, ^57^ It has been well‐accepted that mitochondria form a complex crosstalk network for these cellular functions via the generation of signaling molecules such as mitochondrial DAMPs and metabolic intermediates.^58^ Therefore, changes in the dynamics of molecules located at MAMs have an extremely high likelihood of affecting the status of mitochondria. Proteomic profiling of cardiac MAMs in models of sepsis with and without Beclin‐1 activation will provide a more profound understanding of the role of MAMs in sepsis‐induced cardiomyopathy and their potential regulation by Beclin‐1.

Lastly, a delicate balance is required to control the level of MAMs as well as the space between mitochondria and ER that supports proper mitochondrial function. Animal models of obesity and clinical samples from Alzheimer's patients showed that an excessive induction of MAMs under these disease conditions is pathological, responsible for calcium overload in mitochondria and leading to compromised mitochondrial oxidative capacity and augmented oxidative stress.^21^, ^59^ As summarized in this report, we detected that endotoxemia induces a severe reduction in the mass and function of cardiac MAMs, a response that is maladaptive because it is associated with a deterioration in mitochondria and dysfunction in the heart.^27^ These results indicate that impairments of MAMs are pathological during sepsis‐induced cardiomyopathy. We also obtained data suggesting a new function for Beclin‐1 in supporting the properties of MAMs, either by inducing the new formation of MAMs or by defending them from degradation, which thus improves cardiac mitochondria and protects the heart in response to endotoxemia. In the future, investigations to directly examine the role of MAMs in sepsis outcomes and to determine whether MAMs are essential for Beclin‐1‐dependent cardiac protection will unveil new knowledge about the biological function of MAMs and improve the current understanding of the Beclin‐1‐dependent signaling network in the heart during sepsis.

An immediate question following our new findings reported here is whether and how Beclin‐1 initiates the formation of MAMs in the heart. In this regard, we suspect that Beclin‐1 may achieve this function via the mitochondrial GTPase mitofusin 2 (Mfn2). Mfn2 was originally identified as one of the mitochondria fusion factors but is now recognized as a key regulator in the formation of MAMs.^60^, ^61^ This protein is located at MAMs and the mitochondrial outer membrane, and physically interacts with other mitofusins or itself to bring mitochondria and the ER together.^62^, ^63^ Mfn2 is robustly expressed in the heart,^60^ and a deficiency in Mfn2 expression causes fewer MAMs and more gap space between mitochondria and the ER.^64^ In a preliminary study, we detected that LPS decreased the amount of Mfn2 in MAMs, and Beclin‐1 is needed to keep up its level (data not shown). One of our ongoing investigations is aimed at determining the potential signaling pathway between Beclin‐1 and Mfn2 in cardiac MAMs and the responses of these signaling components to endotoxemia.

## AUTHOR CONTRIBUTIONS

Q.S.Z. conceived the project, designed the study, and wrote the manuscript. Y.S., Y.C., S.Q., and H.C. conducted all the experiments. Q.S.Z., Y.S., and Y.C. contributed to the data analysis and interpretation. All the authors approved the final draft.
